# Supplementary material for: Aedes albopictus is present in the lowlands of southern Zambia
Source: Acta Trop. Author manuscript; Available in PMC 2024 Nov 25. (PMC11586621; doi:10.1016/j.actatropica.2023.107115)

SUPPLEMENTARY MATERIAL for: *Aedes albopictus* is present in the lowlands of southern Zambia

Daniel R. Matute and Brandon S. Cooper

TABLE S1. Genbank accession numbers for the COI barcodes used to generate an *Ae. albopictus* gene genealogy.

**Code in Figures 2 and S1**	**Gene Bank accession number**
Aedes aegypti	MK265729
Thailand1	KM613129.1
Thailand3	KM613121.1
Thailand2	KM613128.1
Vietnam3	HQ398902.1
Siavonga1	TBD
Mozambique1	LC726387.1
Mozambique2	LC726393.1
Siavonga2	TBD
Mozambique3	LC726394.1
Mozambique4	LC726392.1
Mozambique5	LC726380.1
Borneo	MN540323.1
Borneo2	MN540322.1
Spain	KU319448.1
Portugal3	MK995330.1
Portugal2	MK995331.1
Mexico1	MT999274.1

Mexico2	MT552470.1
Montenegro	MK505589.1
Spain2	KU319443.1
Portugal1	MK995332
DRC3	MT345383
Morocco2	KU522419
Morocco1	KU522421
DRC2	MT345388
China3	KX886337
China1	KX981869
China2	KX981868
Cameroon1	MH921572
Cameroon3	MH921568
Cameroon4	MH921571
Cameroon2	MH921570
Madagascar2	JN406732
Madagascar3	JN406725
Reunion1	JN406663
Vietnam2	KX573911
Vietnam1	KX495925
ST1	JF309319
ST2	JF309319
ST3	JF309318
DRC1	MT345390
Reunion2	AJ971012
Rep. Congo	MH025948

Madagascar1	AJ971007
Reunion2	AJ971013

FIGURE S1. Maximum likelihood *Ae. albopictus* tree derived from the *COI* mtDNA barcode. We retained all the branches in spite of some low-support branches. Values above each node correspond to the bootstrap support. Branch lengths are proportional but the topology is identical to the one shown in Figure 2.

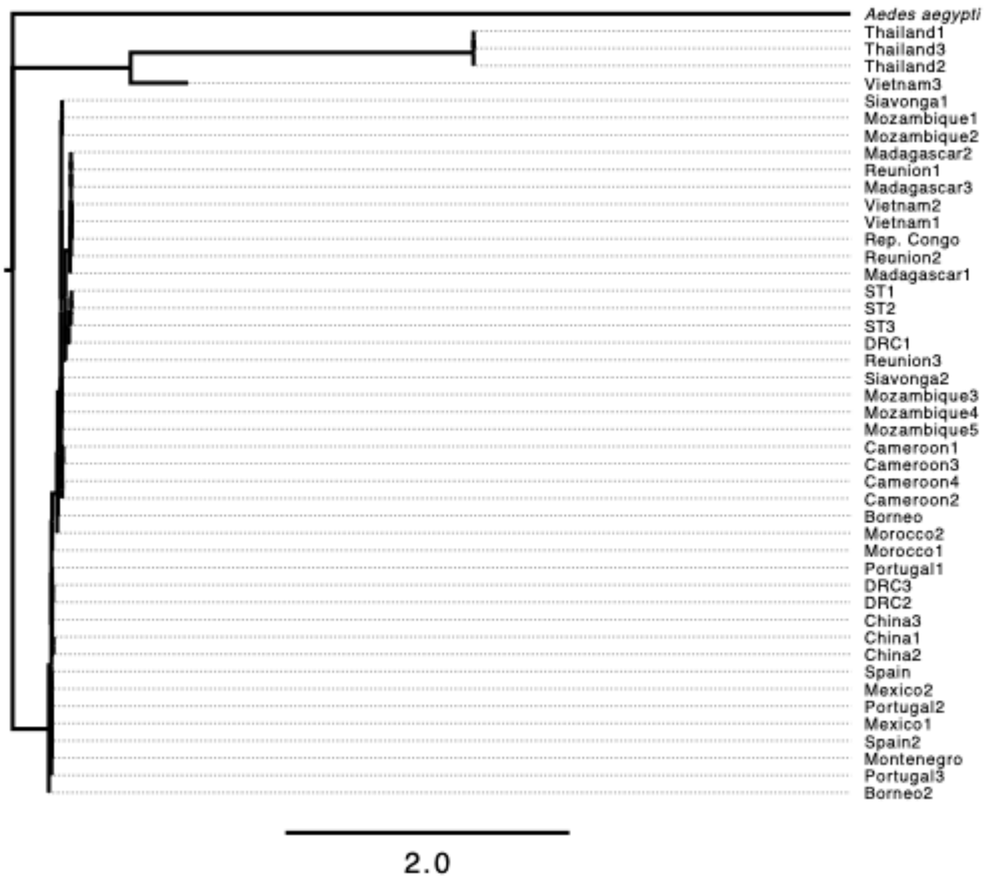

Supplement: Table S1, FIgure S1 [file NIHMS2034535-supplement-Table_S1__FIgure_S1.pdf]
